# Typhoid Fever and Salicylic Acid

**Published:** 1883-01

**Authors:** 

**Affiliations:** Professor at the Academy of Medicine, Paris


TYPHOID FEVER AND SALICYLIC ACID.

In presenting to the Academy of Medicine a work of Dr. Hallspeare on the treatment of typhoid fever by the association of calomel, salicylate of soda, and sulphate of quinine, M. Vulpian asked if against this terrible scourge it would not be possible to employ an antiseptic, slightly soluble, susceptible of arriving, without alteration, in the intestine, and there neutralizing the typhoid virus?

These first trials have been made with iodoform, in which there is no antiseptic property, because, when it is added to a putrid liquid, it neither kills the microbes nor destroys its fetidness.

The salicylate of bismuth, a substance slightly soluble, has not given very satisfactory results. It has not checked the disease, in spite of its prompt antiseptic action, and in doses of eight or ten grammes per day it has only produced a slight lessening of the temperature, with a notable disinfection of the stools. The disease has constantly followed its fatal course, while with a majority of the fever patients have come on the scene symptoms of excessive dyspnœa and nasal or intestinal hemorrhage.

The phenate of soda, given nine grammes per day, although well endured, has led to no sensible modification of the local or general condition.

Boracic acid, given progressively twelve grammes in twenty-four hours, and perfectly acceptable to the patients, by dissolving it in a quart of lemonade, has exerted no salutary influence.

In presence of these results, M. Vulpian seeks a new method.

Leaving aside the neutralization of the poison in its place, wherever that may be, he recalls the fact that typhoid fever, the same as the small-pox, the measles, the scarlet fever, consists in reality of an intoxication caused by the virus absorbed, and which, on its first attack, he seeks to combat in the blood itself and in its organic elements.

The medicine ought to reach not only the microbes, but the nervous centres, which impel the general circulation.

To effect this, his choice is salicylic acid, to which numerous German, Italian and American works have for a long time accorded an action certain and preponderate.

The dose of salicylic acid, given in unleavened bread, is about half a gramme every half hour or hour, but it has been increased successfully to six, ten and twelve grammes.* It is the medium dose of six to seven grammes per day which should form the base of the new medication.

From a careful study of various cases at the Hotel-Dieu, it is found that but little inconvenience is experienced in administering salicylic acid. It has produced in some young persons a little salicylism cerebral, but the delirium did not resemble altogether that of typhoid, and, moreover, it ceased when the medicine was stopped. Symptoms of albuminuria sometimes appeared, but it was impossible to say whether they were caused by the fever or the remedy.

On the other side, the beneficial effects of salicylic acid have always been very striking:

First. The regular and permanent lowering of the temperature from 40.5 degrees to 39 degrees, 38.5 degrees Cent., at the end of twenty-four hours.

Second. Amelioration of the general condition, evident to the physician, appreciable by the acquaintances of the patient, who himself was conscious of it.

Third. Successive oscillations of the thermometer, in direct accord with the administration of salicylic acid, and with its suspension or suppression.

The action of this medicine is, then, logical, though it may not be all-powerful and veritably curative.

Salicylic acid, given in sufficient doses, is, up to this time, one of the most powerful agents in moderating typhoid fever.

This point established, M. Vulpian demanded "if salicylic acid could not be employed as a prophylactic and preventive agent in epidemics of typhoid fever, and if taking daily a moderate dose of this medicine would not have the effect of annihilating the action of the typhoid poison?"—*By M. Vulpian, Professor at the Academy of Medicine, Paris. Translated for Hall's Journal of Health.*

---

*One gramme is equal to twenty-three grains.

